# A Retrospective Analysis of the Association Between Prediabetes and Biomarkers of Intestinal Permeability in Patients with Prediabetes from Durban, South Africa

**DOI:** 10.3390/ijms27198674

**Published:** 2026-09-28

**Authors:** Luyanda Mthethwa, Andile Khathi

**Affiliations:** School of Medicine, College of Health Sciences, University of KwaZulu-Natal, Durban 4041, South Africa; 221028011@stu.ukzn.ac.za

**Keywords:** type 2 diabetes mellitus (T2DM), prediabetes, intestinal permeability, leaky gut

## Abstract

Type 2 diabetes mellitus (T2DM) is a metabolic disorder characterized by insufficient insulin production and diminished insulin receptor sensitivity. Prediabetes, which often precedes T2DM, involves elevated blood glucose levels that do not yet meet T2DM diagnostic criteria. Research has shown that complications linked to T2DM often begin during this prediabetic phase. In this study, we examined changes in intestinal barrier integrity during prediabetes by measuring biomarkers associated with intestinal permeability. With ethical approval, samples were collected from King Edward Hospital and Albert Luthuli Central Hospital in Durban and categorized into type 2 diabetes (T2D), prediabetes (PD), and non-prediabetes (NPD) groups. Blood levels of neutrophils and HbA1c were measured and analyzed, followed by serum zonulin and plasma levels of markers, including IFABP, sCD14, TNF-α, IL-6, glucose, insulin, and pancreatic lipase, which were measured and analyzed. The results showed significantly higher levels of these markers in the PD group compared to the NPD group, with further elevations in the T2D group. These findings align with existing research suggesting an association between increased intestinal permeability and the development of prediabetes. Altogether, these results indicate that prediabetes is a transitional phase marked by metabolic and intestinal permeability changes, though less severe than in T2DM. This underscores the need for additional research to cover a wider range of metabolic health, oxidative stress, inflammation, and vascular function markers, as such comprehensive assessments could provide deeper insight into underlying physiological processes.

## 1. Introduction

The small intestine plays an essential role in the digestive system. It is a crucial site for the enzymatic digestion of nutrients and the absorption of water [1] and a host to a multitude of gut flora, which play a role in nutrient metabolism and maintaining the integrity/stability of the intestinal wall [2]. The intestinal wall and epithelial cells function to block the movement of intestinal antigens, toxins, and incompletely digested nutrients from entering the blood circulation [3]. Studies have shown that factors such as diets consisting of high amounts of refined carbohydrates and saturated fats, obesity, sedentary lifestyles, and age; all these factors lead to the dysregulation of the gut microbiota, thus affecting the intestinal barrier and leading to leaky gut [3].

In a “leaky gut,” increased intestinal permeability allows luminal antigens, bacterial lipopolysaccharides (LPS), and other harmful substances to pass into the blood circulation [4]. The translocation of these substances triggers an immune response defined by the increase in immune cells such as neutrophils and macrophages, and the release of pro-inflammatory cytokines such as IL-6, IFN-γ, TNF-α, and CRP [5]. The consistent entry of pathogens due to the leaky gut causes chronic inflammation in organs such as the pancreas, causing an increase in insulin secretion [6]. High levels of insulin over a prolonged period lead to insulin resistance and elevated blood glucose levels. If the high levels of blood glucose are maintained for a prolonged period, this results in the development of type 2 diabetes mellitus [7]. However, in most cases, the onset of type 2 diabetes mellitus is preceded by a state referred to as prediabetes.

Intestinal permeability can be evaluated using several circulating biomarkers that reflect different aspects of gut barrier function. Zonulin is a physiological modulator of intestinal tight junctions and is commonly used as an indirect marker of increased intestinal permeability [8]. Intestinal fatty acid-binding protein (I-FABP) is a cytosolic protein released into circulation following enterocyte injury and is therefore considered a marker of intestinal epithelial damage [9]. Monocytes and macrophages release soluble cluster of differentiation 14 (sCD14) in response to lipopolysaccharide (LPS) exposure and are widely recognized as a marker of microbial translocation and systemic immune activation [10]. Alterations in these biomarkers have been associated with obesity, insulin resistance, prediabetes, and T2DM, suggesting that impaired intestinal barrier function may contribute to metabolic disease progression.

Prediabetes is a term that refers to an intermediate phase between normal glucose tolerance (NGT) and full T2DM. In this state, plasma glucose concentrations are elevated above normal but not high enough for a T2DM diagnosis [11]. According to the American Diabetes Association (ADA), prediabetes is defined by one or more of the following criteria: fasting plasma glucose (FPG) of 5.6–6.9 mmol/L (100–125 mg/dL), 2 h plasma glucose of 7.8–11.0 mmol/L (140–199 mg/dL) during a 75 g oral glucose tolerance test (OGTT), or glycated hemoglobin (HbA1c) levels of 5.7–6.4% [12]. Individuals with prediabetes are at increased risk of developing T2DM and associated metabolic complications [13]. Numerous studies have shown that many complications associated with T2DM begin during the prediabetic phase. Research in animals has shown a link between increased intestinal permeability and the onset of prediabetes [14]. However, no studies have yet examined this association in humans.

A meta-analysis conducted in 2021 estimates that 16% of South Africans are affected by prediabetes, with KwaZulu-Natal Province reporting the second-highest prevalence rates [15]. Research in Durban, South Africa, indicates a concerning average prediabetes prevalence of 68% across ADA and WHO criteria [15]. This makes Durban a relevant location for examining physiological changes associated with prediabetes. Accordingly, this study seeks to explore changes in leaky gut markers among individuals with prediabetes in this population.

## 2. Results

Significant differences were observed in all intestinal permeability, inflammatory, and metabolic biomarkers across the three study groups. Prediabetic participants consistently exhibited values intermediate between those of normoglycaemic controls and individuals with T2D. Markers of intestinal permeability (zonulin, IFABP, and sCD14), systemic inflammation (TNF-α and IL-6), and glucose metabolism (fasting glucose, insulin, HbA1c, and HOMA-IR) demonstrated a progressive increase from ND to PD to T2D, reflecting the gradual deterioration of intestinal barrier integrity, inflammatory status, and metabolic regulation along the glycaemic continuum. Neutrophil concentrations, however, exhibited a different pattern, with reduced levels observed in the PD group. Correlation analyses revealed significant positive associations between intestinal permeability biomarkers, inflammatory markers, and measures of glycaemic control, indicating that worsening glucose metabolism is accompanied by increased intestinal permeability and systemic inflammation.

### 2.1. Intestinal Permeability Markers

**Figure 1:**
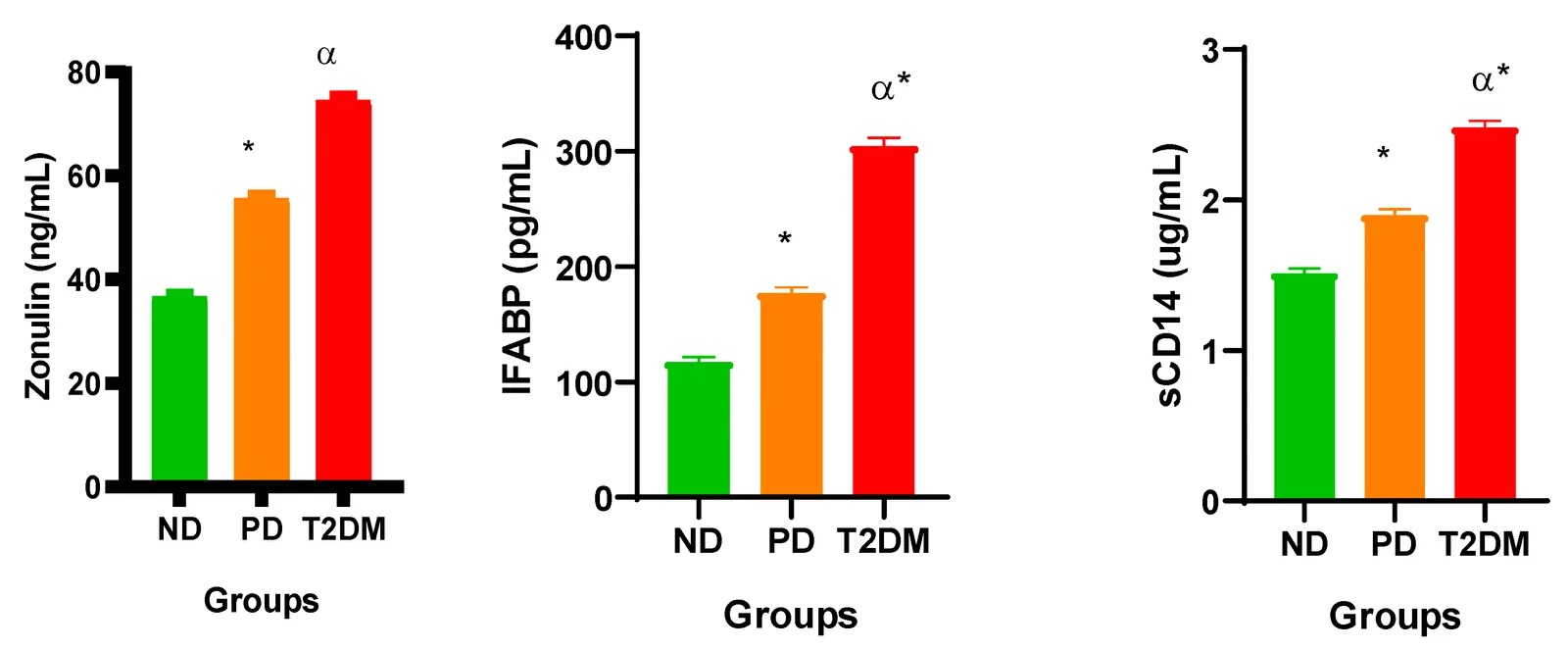
Intestinal permeability marker concentrations in the non-diabetic (ND) group, prediabetic (PD) group, and type 2 diabetic (T2D) group. Values are presented as means ± SEM (*n* = 135); * indicates *p* ˂ 0.0001 against ND, and α indicates *p* ˂ 0.0001 against PD.

**Figure 2:**
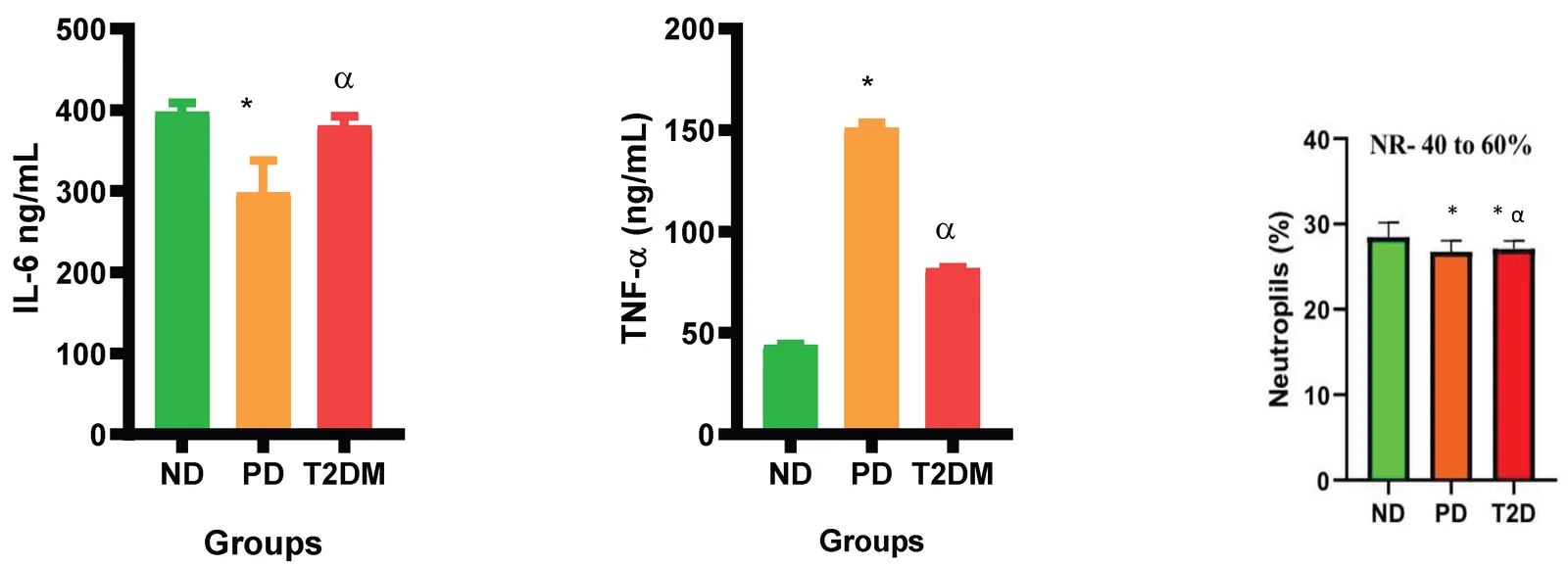
Inflammatory marker concentrations in the non-diabetic (ND) group, prediabetic (PD) group, and type 2 diabetic (T2D) group. Values are presented as means ± SEM (*n* = 135); * indicates *p* ˂ 0.0001 against ND, and α indicates *p* ˂ 0.0001 against PD.

**Figure 3:**
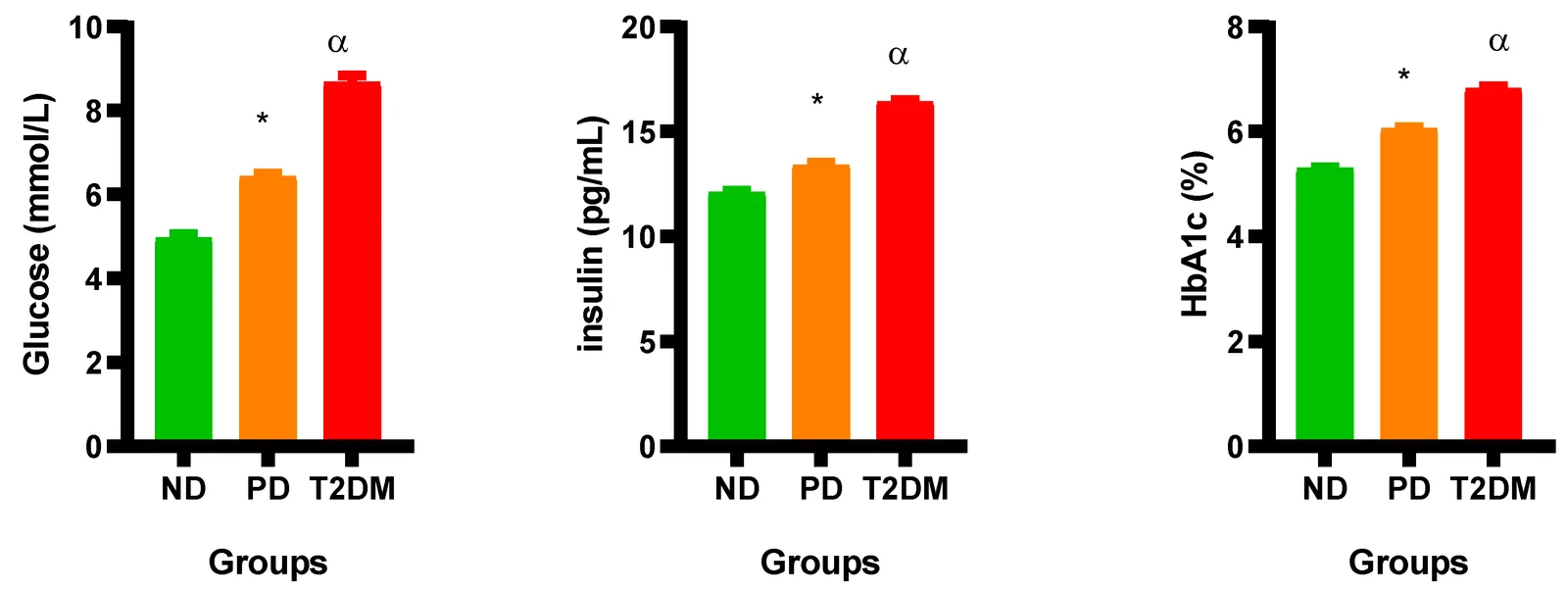
Metabolic marker concentrations in the non-diabetic (ND) group, prediabetic (PD) group, and type 2 diabetic (T2D) group. Values are presented as means ± SEM (*n* = 135); * indicates *p* ˂ 0.0001 against ND, and α indicates *p* ˂ 0.0001 against PD.

Markers of intestinal permeability showed a progressive increase across the glycaemic spectrum from ND to PD and T2D (Figure 1). Serum zonulin concentrations were significantly elevated in the PD group compared with the ND group (*p* < 0.0001), with a further increase observed in T2D participants (Figure 1). Similar trends were evident for IFABP and sCD14, with both markers significantly increased in PD individuals relative to ND controls and further increased in T2D subjects (*p* < 0.0001 for all comparisons; Figure 2 and Figure 3). These findings suggest a gradual deterioration in gut barrier integrity and increased microbial translocation as diabetes progresses from normoglycaemia.

### 2.2. Inflammatory Markers

Markers of systemic inflammation differed significantly among the study groups (Figure 2). Neutrophil concentrations were significantly lower in the PD group than in the ND group, but increased again in T2D participants. Pro-inflammatory cytokines also displayed marked alterations. TNF-α concentrations were elevated in both dysglycaemic groups, with the highest levels observed in T2D participants. Similarly, IL-6 concentrations increased progressively across groups, with significantly higher values observed in T2D subjects than in PD participants. Collectively, these findings indicate activation of inflammatory pathways during the development of prediabetes and type 2 diabetes.

### 2.3. Metabolic Markers

As expected, markers of glycaemic control increased progressively from ND to PD and T2D participants (see Figure 3). Plasma glucose concentrations were significantly elevated in PD individuals compared with ND controls and further increased in T2D patients. A similar pattern was observed for insulin concentrations, suggesting increasing insulin resistance across the groups. HbA1c concentrations also rose progressively, confirming worsening long-term glycaemic control from prediabetes to overt diabetes.

### 2.4. Pancreatic Marker

**Figure 4:**
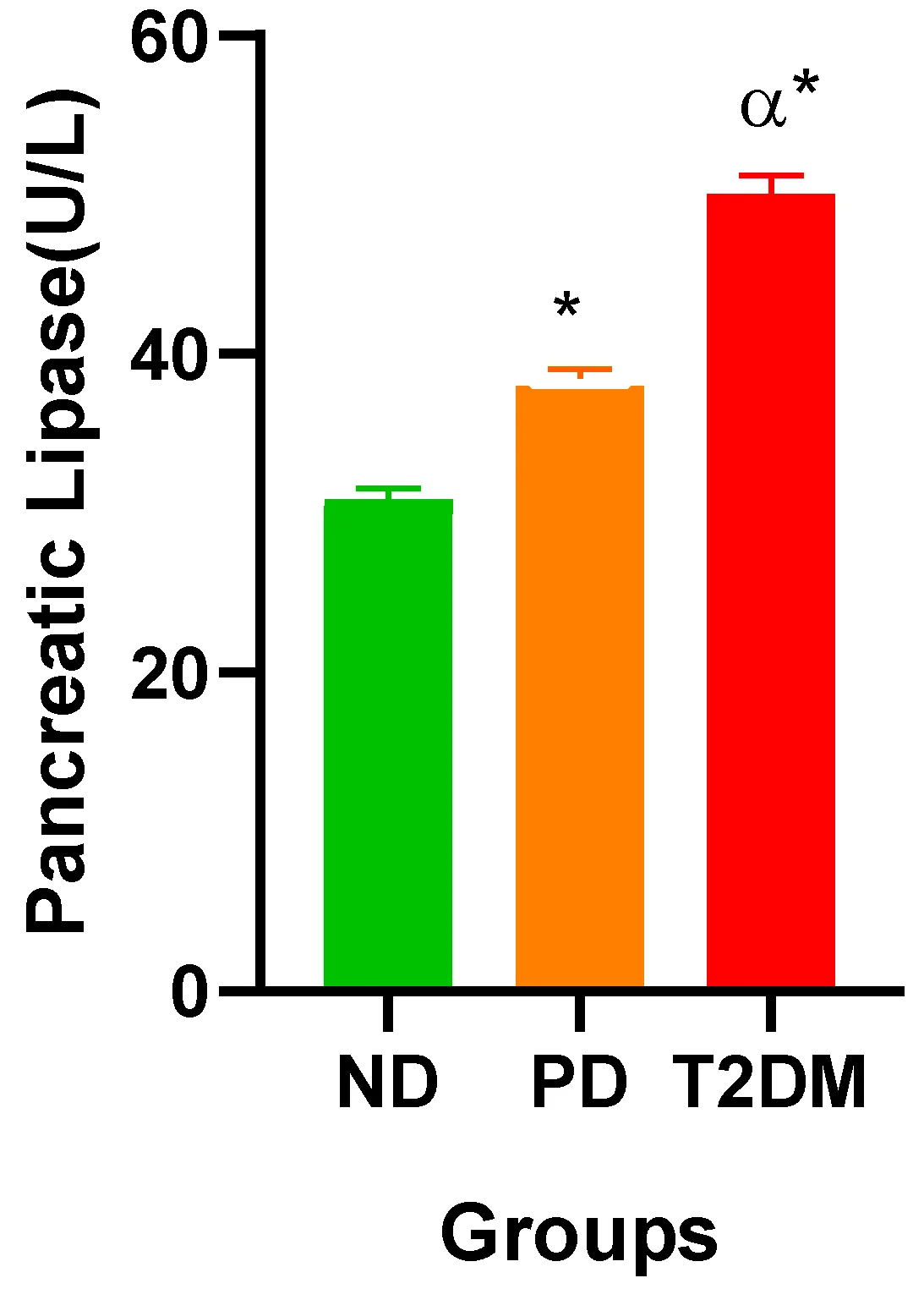
Plasma Pancreatic lipase concentrations in the non-diabetic (ND) group, prediabetic (PD) group, and type 2 diabetic (T2D) group. Values are presented as means ± SEM (*n* = 135); * indicates *p* ˂ 0.0001 against ND, and α indicates *p* ˂ 0.0001 against PD.

Plasma pancreatic lipase concentrations were significantly higher in PD participants than in ND controls and increased further in the T2D group (see Figure 4). This finding suggests that pancreatic alterations may already be present during the prediabetic stage and become more pronounced with disease progression.

## 3. Discussion

The small intestine plays a critical role in nutrient digestion and absorption while maintaining intestinal barrier integrity through its interactions with the gut microbiota [1,4]. Disruption of gut microbial balance by factors such as unhealthy diets, obesity, physical inactivity, and aging can increase intestinal permeability, allowing the translocation of bacterial products and other luminal antigens into the circulation [4]. This process promotes systemic inflammation, which has been implicated in the development of insulin resistance and the progression of type 2 diabetes mellitus (T2DM) [6]. Prediabetes represents an intermediate stage between normal glucose regulation and T2DM and is associated with an increased risk of metabolic complications. Given the high prevalence of prediabetes in South Africa, particularly in KwaZulu-Natal, this study investigated biomarkers of intestinal permeability, inflammation, and metabolic dysfunction in individuals with prediabetes to better understand their potential role in disease progression.

Zonulin is a protein that regulates the permeability of the tight junctions between cells in the intestinal epithelium [16]. Zonulin plays a critical role in maintaining the integrity of the gut barrier by controlling the opening and closing of these junctions, preventing the translocation of harmful substances from the gut lumen into the bloodstream [17]. This process is essential for ensuring a selective barrier that allows the absorption of nutrients while keeping pathogens and toxins out [17]. In people with T2DM, elevated zonulin levels have been associated with heightened intestinal permeability, often termed “leaky gut.” [18]. This heightened permeability allows harmful substances, such as bacterial lipopolysaccharides, to pass into the bloodstream, which triggers systemic inflammation and plays a role in the development of insulin resistance [19]. The compromised gut barrier, regulated by zonulin, is thought to worsen the metabolic inflammation observed in T2DM, further reducing insulin sensitivity and accelerating disease progression [20]. As a result, elevated zonulin levels in T2DM are associated with both gut barrier dysfunction and the metabolic complications that contribute to insulin resistance (see Table 1) [21]. To our knowledge, this study is the first to investigate changes in serum zonulin levels in the prediabetic state among an African population, particularly within the South African context. Serum zonulin levels in the T2D group were noticeably higher than those in the ND group in this investigation. In care, the difference was less pronounced in prediabetes than in T2DM, where, in contrast to the T2D group, the PD group’s serum zonulin levels were much lower.

The increase in zonulin in prediabetic individuals is believed to cause a mild increase in intestinal permeability, leading to the translocation of microbial products, such as lipopolysaccharides (LPS), into the bloodstream (see Table 1) [14]. This triggers a low-grade inflammatory response that can contribute to insulin resistance, a hallmark of prediabetes [22]. However, the degree of zonulin elevation and the resulting permeability are less pronounced in prediabetes than in T2DM, where gut barrier dysfunction is more severe (see Table 2). In prediabetes, the pancreas is still able to compensate for the initial stages of insulin resistance by producing more insulin, but the elevated zonulin levels signal the beginning of a dysregulated gut-immune axis that can worsen as the condition progresses toward T2DM [19].

IFABP is a small cytosolic protein found predominantly in enterocytes of the small intestinal mucosa [23]. IFABP functions as a high-affinity intracellular lipid-binding molecule that facilitates the uptake and trafficking of long-chain fatty acids within enterocytes, thereby contributing to intestinal lipid metabolism and overall energy homeostasis [23]. IFABP plays a critical role in maintaining gut barrier integrity, and elevations in its plasma concentration serve as a sensitive indicator of compromised intestinal epithelial function [24]. Increased circulating IFABP reflects the breakdown of epithelial tight junctions and a subsequent rise in intestinal permeability, allowing luminal antigens and microbial products to translocate into the systemic circulation, where they contribute to the development and amplification of systemic inflammation [25]. IFABP is elevated in conditions like T2DM, where elevated IFABP correlates with worse metabolic markers and longer duration of hyperglycemia, indicating progressive intestinal barrier dysfunction [24]. Indeed, in the current study, IFABP concentrations were significantly higher in the T2DM group than in the ND group, suggesting intestinal epithelial damage and alterations in the microbiota [25].

Prediabetes is characterized by low-grade inflammation, early gut barrier dysfunction, and metabolic stress, such as early hyperglycemia. No studies to date have directly measured IFABP levels in individuals with prediabetes; however, evidence from early hyperglycemia cohorts shows that IFABP levels begin to rise with the onset of metabolic dysregulation, suggesting that intestinal barrier impairment likely starts during the prediabetic state [24]. To the best of our knowledge, this study is the first to investigate alterations in IFABP concentrations during the prediabetic state. In the current study, the results show a significant increase in IFABP concentrations in the PD group when compared to the ND group, reflecting early intestinal epithelial stress. Research shows that prediabetes is associated with diet-induced changes in gut microbiota and the development of a “leaky gut,” where the intestinal barrier becomes compromised before full-blown type 2 diabetes emerges [4]. This impaired barrier allows greater exposure of enterocytes to luminal antigens and inflammatory mediators, creating a state of low-grade inflammation that damages the intestinal mucosa [26,27]. The increase in IFABP levels in the PD correlates with the increase in zonulin levels in this current study; when zonulin increases, tight junctions loosen, increasing intestinal permeability [8,28]. This heightened permeability exposes enterocytes to inflammatory and microbial stress, leading to epithelial injury. IFABP, released from damaged enterocytes, rises as a result [29,30]. Therefore, elevated zonulin and IFABP together may reflect both functional barrier breakdown and actual cellular injury.

sCD14 is the circulating form of CD14, a pattern-recognition co-receptor primarily expressed on monocytes, macrophages, neutrophils, and dendritic cells [31]. Its physiological role extends beyond classical microbial sensing, contributing to metabolic inflammation by activating monocytes and adipocytes and driving the downstream release of pro-inflammatory cytokines [32]. Individuals with type 2 diabetes and those progressing toward diabetes often exhibit elevated sCD14 levels, though large cohort studies show that the predictive value of sCD14 for incident diabetes is attenuated after adjustment for conventional risk factors. Indeed, the current study shows a significant increase in the T2DM group compared with the ND group. Elevated sCD14 has been observed in individuals with established insulin resistance and T2DM, consistent with a state of chronic metabolic inflammation [32]. Evidence from a recent study indicates that sCD14 is associated with insulin resistance and that, in the prediabetic state, sCD14 levels demonstrate an association with insulin resistance [32]. To the best of our knowledge, this study is the first to investigate alterations in circulating sCD14 levels during the prediabetic state. The results of the current study showed a significant increase in the levels of sCD14 in the PD group when compared to the ND group, further suggesting an association with insulin resistance. However, this relationship becomes non-significant when controlling for other inflammatory markers, indicating that sCD14 is not an independent driver of early dysglycemia [32]. sCD14 and IFABP are both indicators of impaired intestinal barrier function, but each represents a different component of the process [33]. IFABP rises when enterocytes are damaged, releasing this cytosolic protein into circulation [34], while sCD14 increases in response to microbial translocation, indicating immune activation triggered by LPS and other gut-derived antigens crossing the compromised barrier [34], thus providing strong combined evidence of gut barrier breakdown and the passage of bacterial components into circulation.

Neutrophils (PMNs) play a central role in innate immunity, contributing to both pathogen defense and tissue repair [35]. However, this study identified a consistent reduction in circulating PMN levels across all groups assessed. Several factors may account for this decline, including potential disruptions in bone-marrow-derived neutrophil production or maturation, possibly influenced by the unique nutritional landscape of KwaZulu-Natal, where undernutrition and overnutrition occur simultaneously [36]. This environment suggests that even individuals without T2DM may display altered neutrophil profiles driven by dietary and environmental pressures [36]. Additional contributors, such as chronic gastrointestinal inflammation or exposure to immunotoxic agents, may further exacerbate neutropenia [36]. In T2DM, diminished PMN counts align with the established pattern of neutrophil migration from the bloodstream into chronically inflamed tissues, including adipose and vascular compartments [37].

The present findings indicate that this redistribution may begin as early as the prediabetic state. Notably, despite the pro-inflammatory nature of hyperglycemia and hyperlipidemia conditions that would typically enhance neutrophil production, circulating PMN levels nevertheless continue to fall [38]. Overall, these observations mirror the disruptions seen with sCD14, suggesting that both markers capture early innate immune alterations that arise during the transition from normoglycemia to prediabetes and ultimately to T2DM, even though neither appears to independently drive early metabolic deterioration.

TNF-α is a pro-inflammatory cytokine produced primarily by macrophages and other immune cells in response to infection or injury [39]. Under normal physiological conditions, TNF-α plays a crucial role in regulating immune responses, promoting the inflammatory process to help fight infections, and facilitating tissue repair by inducing apoptosis and promoting cell survival [40]. In T2DM, increased levels of TNF-α are linked to ongoing low-grade inflammation that plays a role in insulin resistance [41]. TNF-α disrupts insulin signaling by enhancing the serine phosphorylation of insulin receptor substrate-1 (IRS-1), which diminishes its capacity to activate the downstream signaling pathways necessary for glucose uptake [42]. Indeed, in the present study, patients with T2D had significantly higher TNF-α levels than those in the ND group. Furthermore, TNF-α levels in the PD group were significantly elevated compared with the ND group, although they remained lower than in the T2D group. This can be clarified by the fact that in prediabetes, the elevated TNF-α levels can impair insulin signaling by promoting serine phosphorylation of insulin receptor substrate-1 (IRS-1), which diminishes the effectiveness of insulin in facilitating glucose uptake in tissues [42]. This leads to a gradual increase in insulin resistance, a hallmark of prediabetes, as the body attempts to compensate by producing more insulin [42]. However, the increase in TNF-α is typically less severe in prediabetes than in T2DM, where persistent high levels of TNF-α contribute to more significant metabolic dysfunction and beta-cell failure [41] (see Table 2). Significantly, the participants with prediabetes in our study exhibited increased levels of zonulin, suggesting increased intestinal permeability that leads to the entry of pathogens that induce an inflammatory response.

IL-6 is a cytokine that plays a multifaceted role in immune responses, inflammation, and metabolic functions [43]. Under normal physiological conditions, various cell types, including macrophages, T cells, and fibroblasts, produce IL-6, which is crucial for the acute-phase response to infections or injuries. It stimulates the production of acute-phase proteins and promotes B-cell differentiation [43]. Additionally, IL-6 impacts glucose metabolism by affecting insulin sensitivity and energy balance [44]. In T2DM, elevated IL-6 levels are associated with chronic low-grade inflammation, which contributes to insulin resistance [44]. Studies have shown that patients with T2DM have higher IL-6 levels than those without T2DM [45]. Our findings support this, revealing significantly higher IL-6 levels in the T2D group than in the ND group. Furthermore, the PD group exhibited notably higher IL-6 levels than the ND group, yet these remained significantly lower than those in the T2D group. In healthy individuals, IL-6 is produced in response to infections or injuries as part of the acute-phase inflammatory response, helping to regulate immune functions and promote tissue repair [46]. However, in prediabetic individuals, the body experiences early metabolic dysregulation and insulin resistance, leading to a moderate increase in IL-6 levels. The increase in IL-6 in prediabetes reflects the body’s attempt to manage insulin sensitivity and maintain glucose homeostasis. In contrast, in individuals with T2DM, IL-6 levels are significantly higher due to a more advanced state of chronic inflammation. Furthermore, the findings of this study indicate increased IL-6 levels in the PD group. This aligns with elevated levels of TNF-α, indicating an inflammatory response due to leaky gut.

Insulin is a peptide hormone produced by the beta cells of the pancreas in response to elevated blood glucose levels, primarily after eating. Its primary function is to regulate glucose homeostasis by facilitating the uptake of glucose into muscle and adipose tissues, and by inhibiting hepatic glucose production [47]. Insulin binds to its receptor on target cells, activating a signaling cascade that promotes glucose transport into the cells via glucose transporter proteins like GLUT4 [48]. This process ensures that blood glucose levels remain within a healthy range and that excess glucose is stored as glycogen or used for energy. In T2DM, insulin resistance occurs when the body’s cells do not respond properly to insulin, prompting the pancreas to produce more insulin [49]. Over time, this increased demand can lead to beta-cell dysfunction, resulting in insufficient insulin production and persistently elevated blood glucose levels [50]. The combination of insulin resistance and impaired insulin secretion drives the progression of T2DM and its associated complications [51].

In the present study, T2D patients presented with significantly higher levels of insulin than the ND group. Patients with PD, on the other hand, showed significantly lower insulin levels than T2D patients, consistent with previous research. Prediabetic individuals often exhibit insulin resistance, in which cells in tissues such as muscle, liver, and adipose tissue do not respond effectively to insulin, reducing glucose uptake and leading to higher blood glucose levels [51]. To compensate for this reduced sensitivity, the pancreas secretes elevated levels of insulin (hyperinsulinemia) to maintain normal or near-normal blood glucose levels. However, insulin levels in prediabetes are typically lower than in individuals with T2DM, as the pancreas can still produce sufficient insulin to overcome insulin resistance, though this compensatory mechanism is not as severely strained as in full-blown diabetes [49,52]. Over time, persistent insulin resistance and elevated insulin demands lead to beta-cell dysfunction, which gradually impairs the pancreas’s ability to produce insulin efficiently [51]. In prediabetes, this dysfunction has begun but is not yet as severe as in T2DM, resulting in insulin levels that are higher than normal but not as drastically elevated as in T2DM. The reduced insulin secretion capacity in prediabetes is enough to prevent significant hyperglycemia, but it indicates the early stages of pancreatic strain that could progress to diabetes if not managed [53,54]. The participants with PD in our study notably showed elevated insulin levels, which aligns with an inflammatory response indicating hyperactivation of the pancreas, leading to high insulin secretion and insulin resistance.

Pancreatic lipase is the primary enzyme responsible for the hydrolysis of dietary triglycerides in the human digestive system [55]. It is synthesized and secreted by the exocrine acinar cells of the pancreas and delivered to the duodenum via the pancreatic duct [56]. The core physiological function of pancreatic lipase is to catalyze the hydrolysis of triglycerides into monoglycerides and free fatty acids, which are sufficiently small to be absorbed by enterocytes in the intestinal lining [56,57]. Studies evaluating exocrine function in T2DM reveal that individuals with the disease commonly exhibit reduced serum and intraluminal pancreatic lipase levels, although long-standing diabetes is commonly associated with reduced pancreatic states [58]. This elevation does not necessarily reflect increased digestive function; instead, it typically reflects stress or irritation of pancreatic tissue, or systemic changes that cause lipase to “spill” into the bloodstream [58]. Indeed, the current study shows a notable increase in pancreatic lipase concentrations in the T2DM group compared with the ND group. In prediabetes, hyperinsulinemia develops as the pancreas compensates for insulin resistance [59]. This state is accompanied by dyslipidemia, inflammation, and metabolic stress, all of which disrupt normal lipid handling and may secondarily influence the exocrine pancreas, potentially increasing pancreatic lipase output or leakage [60]. To the best of our knowledge, studies investigating circulating pancreatic lipase levels during the prediabetic state remain limited, and the present study provides novel evidence of alterations in pancreatic lipase concentrations during early dysglycaemia. The observed increase in pancreatic lipase levels in the PD group compared to the ND group may reflect early metabolic and inflammatory disturbances that precede overt type 2 diabetes (see Table 2). Although chronic T2DM is often associated with reduced exocrine pancreatic output, several studies indicate that circulating pancreatic lipase can rise in earlier dysglycemic states due to subclinical pancreatic stress and enzyme leakage. Evidence also shows that early dysglycemic states are characterized by low-grade pancreatic inflammation, metabolic stress, and structural changes in the pancreas that increase acinar cell permeability, allowing pancreatic lipase to leak into the bloodstream [58].

Glucose serves as a primary source of metabolic energy for tissues [61]. Insulin plays a key role in regulating glucose levels, ensuring a consistent energy supply [62,63]. Insulin resistance occurs when cells fail to respond effectively to insulin signals, impairing glucose uptake from the blood [62,63]. This results in hyperglycemia, eventually leading to T2DM [62,63]. According to the current literature on the metabolic effects of type 2 diabetes, the T2D group in this study had considerably higher glucose levels than the ND group [64]. In contrast, glucose levels in the PD group were higher than in the ND group but lower than in the T2D group. Prediabetes is characterized by insulin-related problems such as insulin resistance and β-cell dysfunction [65]. Tyrosine kinase insulin receptors are disrupted by insulin resistance, which stops intracellular GLUT4 translocation and eventually results in hyperglycemia due to decreased glucose uptake [66]. Participants in the PD group showed elevated glucose levels, which correlate with increased insulin levels indicative of insulin resistance, reflecting a reduced cellular response to insulin and resulting in elevated blood glucose (see Table 3).

The HbA1c test is used to evaluate a person’s glucose control. The test shows the average blood sugar level over the past 90 days as a percentage. The test can also be used to diagnose T2D [21]. The normal HbA1c of a non-diabetic individual is <5.7%, which is less than 2. Multiple studies have shown that HbA1c increases over time in T2D due to chronic hyperglycemia [21]. Indeed, the results presented in this study show a significant increase in insulin concentration in T2D patients compared with NPD patients. In the prediabetic group, we observed a notable increase in insulin compared with the NPD group, and a similar increase when comparing the T2DM group to the PD group. Prediabetes is often seen as an intermediary stage between normal glucose metabolism and full-blown T2D. While HbA1c levels are higher than in ND, they are lower than in T2D because the degree of insulin resistance and hyperglycemia is not as pronounced. In T2D, chronic hyperglycemia leads to significantly higher HbA1c levels due to more sustained periods of elevated blood glucose. The findings of this study corroborate previous studies showing that patients with prediabetes may exhibit insulin concentrations (see Table 3) [67]. Additionally, our study found increased HbA1c in the PD group. During PD, elevated glucose levels increase the amount of glucose bound to hemoglobin in red blood cells, forming glycated hemoglobin (HbA1c). Over time, the higher the blood glucose levels, the more HbA1c is produced, reflecting a longer-term measure of blood sugar control.

## 4. Materials and Methods

### 4.1. Study Design and Sampling

This retrospective study was conducted at the University of KwaZulu-Natal, Westville, South Africa. Ethical approval for this study was obtained from the University of KwaZulu-Natal Research Ethics Committee (ethics number: BE266/19), and consent was obtained for the use, storage, and future use of the samples. Patient samples for this study were recruited from the Inkosi Albert Luthuli Central Hospital and King Edward Hospital. The samples were classified according to the American Diabetes Association (ADA) criteria using glycated hemoglobin (HbA1c) and blood glucose measurements recorded at the time of sample collection. Participants were categorized as non-diabetic (ND) (HbA1c < 5.7%), prediabetic (PD) (HbA1c 5.7–6.4%), or type 2 diabetic (T2D) (HbA1c ≥ 6.5%). Blood glucose concentrations were used to support classification where applicable. A total of 135 participants were included and equally distributed among the ND (*n* = 45), PD (*n* = 45), and T2D (*n* = 45) groups. All participants in the T2DM group were newly diagnosed at the time of recruitment, and blood samples were collected prior to the initiation of insulin therapy or oral hypoglycemic medications. This study included patients of all genders and all ethnicities between the ages of 25 and 45 years. Individuals with high glycated hemoglobin and blood glucose levels were also included in the study. Subjects were excluded from the study if they had a history of liver disease, heart disease, hypertension, blood disorders, HIV positivity, depression, or small bowel disorders. Patients taking medications for the disorders or any other medications that could affect study results were also excluded. The samples were divided based on patients’ blood glucose and glycated hemoglobin levels, which were recorded at hospital admission or at the time of study control recruitment. Glycaemic variables were classified based on the criteria outlined by the American Diabetes Association (ADA). HbA1c levels were measured in the collected blood samples using an enzyme-linked immunosorbent assay (ELISA) kit (Elabscience and Biotechnology, Wuhan, China). An automated Beckman Colter cell counter (Indianapolis, IN, USA) was used to measure neutrophil counts in each blood sample. Following the measurement, the samples were centrifuged at 503 g for 15 min at 4 °C (Eppendorf, Hamburg, Germany). Plasma and serum were then collected and stored at −80 °C in a Bio Ultra freezer (Snijders Scientific, Tilburg, the Netherlands) until biochemical analysis commenced.

### 4.2. Biochemical Analysis

The concentrations of zonulin, tumor necrosis factor (TNF), intestinal fatty acid binding protein (IFABP/FABP2), sCD14, interleukin-6 (IL-6), glucose, and insulin were determined using separate specialized ELISA kits, and pancreatic lipase was determined using a metabolic assay kit, according to the manufacturer’s instructions (Elabscience and Biotechnology, Wuhan, China). Each reaction was performed in a microplate coated with the necessary antibody for each biomarker. Standards and samples were pipetted into the wells to allow target-specific binding with the immobilized antibody. After incubation, unbound components were removed by washing the plate. The microplate was treated with the conjugate, then washed further. Tetramethylbenzidine (TMB) substrate was then added to each reaction to initiate colorimetric development. The absorbance of the colored product was measured to quantify the target antibody.

It should be noted that while established clinical reference ranges exist for biomarkers such as glucose, HbA1c, insulin, and neutrophil counts, no universally accepted reference ranges are currently available for biomarkers including zonulin, IFABP, sCD14, TNF-α, and IL-6. Their interpretation, therefore, depends on the assay used and is generally based on comparisons between study groups rather than on standardized diagnostic thresholds.

### 4.3. Statistical Analysis

All data are presented as the mean ± SEM. Statistical analysis was conducted using GraphPad Prism (version 5.00; GraphPad Software, Inc., San Diego, CA, USA). Normality was assessed using the D’Agostino & Pearson test. One-way ANOVA, followed by the Tukey-Kramer post hoc test, was used for group comparisons as appropriate. A *p*-value < 0.05 was considered statistically significant.

Correlation analyses were conducted using GraphPad Prism. Spearman’s rank correlation coefficient was used to evaluate the associations between intestinal permeability biomarkers, inflammatory markers, and indices of glucose metabolism, including fasting glucose, insulin, HbA1c, and HOMA-IR. The strength and direction of associations were determined using Spearman2019s correlation coefficient (ρ), and statistical significance was defined as *p* < 0.05.

Insulin resistance was estimated using the Homeostatic Model Assessment of Insulin Resistance (HOMA-IR), calculated as fasting insulin (µU/mL) × fasting glucose (mmol/L)/22.5.

## 5. Conclusions

In summary, this study shows clear differences in important metabolic and inflammatory markers between non-diabetic, prediabetic (PD), and T2DM groups. Higher levels of plasma zonulin, IFABP, sCD14, TNF-α, IL-6, insulin, glucose, pancreatic lipase, and HbA1c were observed in both the PD and T2DM groups compared with non-diabetics, with the highest levels in T2DM patients. These results point to a gradual worsening of gut permeability, inflammation, and insulin resistance as individuals progress from prediabetes to T2DM. Although zonulin, IFABP, and sCD14 were lower in the PD group than in T2DM, they still indicate early gut barrier problems and low-grade inflammation that lead to insulin resistance. Additionally, the rise in insulin and HbA1c levels in prediabetes reflects the body’s attempt to control blood sugar, though less effectively than in T2DM. This study highlights the need for early identification and treatment of prediabetes to prevent worsening health and the development of T2DM.

### Limitations and Future Studies

The current study provides insight into the associations among metabolic markers, intestinal permeability, and the development of prediabetes in individuals with varying glycaemic states. However, several considerations could improve the study’s quality and applicability. A sample size larger than 45 could enhance the generalizability of the results, increase the study’s statistical power, and allow for subgroup analysis. Conducting a study with knowledge of the diverse gender population could increase the generalizability of the results, because a previous study has shown that certain hormones can interfere with the production of inflammatory cytokines, especially in females.

Although serum LPS measurements may have provided direct evidence of microbial translocation, LPS has a short half-life in circulation and is rapidly cleared or neutralized, making its measurement challenging. Consequently, this study relied on sCD14 as a surrogate marker of LPS-mediated immune activation. Future studies incorporating both LPS and downstream inflammatory markers may provide a more comprehensive assessment of the relationship between intestinal permeability and systemic inflammation.

A detailed dietary and lifestyle analysis could have been conducted, gathering more detailed data on participants’ diets, exercise habits, sleep patterns, and other lifestyle factors, which could help clarify their impact on gut permeability, inflammation, and insulin resistance. We therefore recommend a longitudinal study that could incorporate these factors as well.

## Figures and Tables

**Table 1:** Correlation analysis for the entire study (Spearman’s rank correlation analysis).

Variables	Correlation Coefficient	*p*-Value
Glucose vs. Zonulin	0.8956	<0.0001
Glucose vs. TNF-α	0.4491	<0.0001
Glucose vs. Pancreatic Lipase	0.7212	<0.0001

**Table 2:** Spearman’s rank correlation analysis for the PD group.

Variables	Correlation Coefficient	*p*-Value
Glucose vs. Zonulin	0.2616	0.0826
Glucose vs. TNF-α	0.1494	0.3273
Glucose vs. Pancreatic Lipase	0.1094	0.4745

**Table 3:** HOMA-IR.

Variable	ND (*n* = 45)	PD (*n* = 45)	T2DM (*n* = 45)	*p*-Value
HOMA-IR	2.667 (2.433–2.970)	3.913 (3.495–4.073)	6.228 (5.572–7.116)	˂0.001

Participants with prediabetes and T2DM exhibited significantly higher HOMA-IR values compared with normoglycaemic participants.

## Data Availability

The original contributions presented in this study are included in the article. Further inquiries can be directed to the corresponding author.

## Abbreviations

The following abbreviations are used in this manuscript:

ADA	American Diabetes Association
AK	Andile Khathi
CRP	C-reactive protein
ELISA	enzyme-linked immunosorbent assay
GRADE	Grading of Recommendations Assessment, Development and Evaluation
HbA1c	glycated hemoglobin
IFG	impaired fasting glucose
IGT	impaired glucose tolerance
IL-6	Interleukin 6
IFABP	intestinal fatty acid binding protein
LM	Luyanda Mthethwa
NPD	non-prediabetes
OGTT	oral glucose tolerance test
PD	prediabetes
sCD14	Soluble CD14
SEM	standard error of the mean
T2D	type 2 diabetes
T2DM	type 2 diabetes mellitus
TNF-α	tumor necrosis factor—alpha
UKZN	University of KwaZulu-Natal
WHO	World Health Organization
